# Dynamic Proteome Landscape During Preimplantation Human Embryo Development and Trophectoderm Stem Cell‐Differentiation

**DOI:** 10.1002/pmic.70017

**Published:** 2025-08-01

**Authors:** Alin Rai, Qi Hui Poh, Hiroaki Okae, Takahiro Arima, Mehdi Totonchi, David W. Greening

**Affiliations:** ^1^ Baker Heart and Diabetes Institute Melbourne Victoria Australia; ^2^ Baker Department of Cardiometabolic Health University of Melbourne Melbourne Victoria Australia; ^3^ Baker Department of Cardiovascular Research Translation and Implementation La Trobe University Victoria Australia; ^4^ Department of Informative Genetics Environment and Genome Research Center Tohoku University Graduate School of Medicine Sendai Japan; ^5^ Department of Genetics Reproductive Biomedicine Research Center Royan Institute for Reproductive Biomedicine ACECR Tehran Iran

**Keywords:** blastocyst, human embryo, proteomics, reproduction, stem cells, trophoblasts

## Abstract

Embryo development involves fertilization of a mature ovum, which, after sequential cell divisions (2‐,4‐8‐cells and morula), undergoes differentiation into implantation competent blastocyst. The blastocyst comprises of inner cell mass surrounded by an outer layer of cells called the trophoblast (TSblast) that, upon attachment to the endometrium, differentiates into extravillous trophoblasts (EVTs) that facilitates embryo invasion into the endometrium for intrauterine embryo development, and syncytiotrophoblast (ST) that form the placenta. Such cellular differentiation stages are critical for embryogenesis and implantation, although the protein expression landscape remains poorly understood in humans. Using quantitative mass spectrometry analysis, we systematically monitored the protein expression landscape and their dynamic regulation between human ovum (M2), 8‐cell embryo, and blastocysts stages, and trophoblast lineage‐specific differentiation into EVTs and ST. Proteins temporally regulated from M2‐8 cell‐blastocyst stage displayed significant enrichment for metabolic protein networks. We specifically identified 156 proteins associated with 8‐cell embryos to blastocyst development, 54 displayed similar correlation at the transcriptomic level including mitochondrial, junction/secretory granule‐associated proteins that carry glycolytic, antioxidant, and telomerase maintenance functions. We reveal a striking lineage‐specific reprogramming of TSblast proteome during fate‐specification. These findings extend our knowledge of the sequential order of protein landscape reprogramming and processes during early human embryogenesis and trophoblast function.

**Summary:**

- Although genomic and transcriptomic studies have provided key understanding of the genetic programs underlying preimplantation embryo development, the protein expression landscape remains unexplored. Here, a quantitative proteomic study of human preimplantation embryo stages reveal a dynamic proteome landscape from M2, 8‐cell, and blastocyst stage, and during trophoblast stem cell (TS) differentiation.
- Identified key factors in early human embryos and lineage‐specific trophoblast proteome profiles, further correlated with transcriptomic analyses.
- This direct proteomic analysis provides a quantitative and temporal analysis of the dynamic protein expression in human embryos during preimplantation development and a powerful resource to enable further mechanistic studies on human trophoblast development and function.

## 1 Introduction

Human embryo development begins with the fertilization of a mature ovum (Metaphase II, M2) to form the zygote, which undergoes sequential mitotic divisions to form eight totipotent blastomeres of the 8‐cell embryo [1, 2]. During early divisions, the embryo is transcriptionally quiescent, with development driven by existing maternal proteins and RNAs [3, 4]. However, during 8‐cell stage, a major event called the embryonic genome activation (EGA) [3, 4] initiates transcription of embryonic genes essential for 8‐cell embryo compaction into a morula, comprising the outer blastomeres with apicobasal surface surrounding the inner non‐polar cells [1, 2, 5]. The first cell‐specification event [6, 7] creates two distinct lineages to form the blastocyst (BLST), where the apical blastomeres differentiates to form the trophectoderm (TE, which later makes the placenta), and non‐polar cells form the pluripotent inner cell mass (ICM, which later makes the foetus) [8, 9, 10, 11].

The preimplantation embryo then undergoes embryo implantation, during which the blastocyst appositions and attaches to the endometrial epithelium [12] via TE and invades into endometrial tissues for intrauterine development [12, 13, 14]. The TE differentiates into three major subpopulations of trophoblast cells with specialized functions [13, 15, 16, 17], namely cytotrophoblast (CT), extravillous cytotrophoblast (EVT), and syncytiotrophoblast (ST). CT cells are an undifferentiated and proliferative population that can differentiate into either EVT cells that invade the decidualized endometrium and remodel the spiral arteries or multinucleated ST cells formed by fusion of CT cells and produce large quantities of placental hormones and other factors to maintain pregnancy [12, 13, 15, 16, 17]. ST cells are directly in contact with maternal blood and mediate the exchange of gases and nutrients. Human trophoblast lineage specification and differentiation are therefore essential for placentation and successful pregnancy [18]. Impaired trophoblast development and function results in pregnancy complications such as miscarriage, preeclampsia, and intrauterine growth restriction [13, 18, 19, 20].

Recent transcriptomics, epigenomics (such as DNA methylation), and higher‐order chromatin structure studies have greatly expanded our understanding of mechanisms that regulate early embryo development [1, 4, 5, 10, 18, 21, 22, 23, 24, 25, 26] and trophoblast development [17, 27, 28, 29, 30]. Proteins are the executors of most biological programs; however, the abundance of proteins cannot be accurately predicted from transcript abundance alone [31, 32]. Indeed, the correlation between RNA expression and protein expression in the same embryo stages is poor in Xenopus [33], zebrafish [34], and mouse [35]. Moreover, the proteome, not the transcriptome, predicted oocyte superovulation, cleavage arrest, and defective blastocyst formation [36]. Mass spectrometry (MS)‐based proteomics is a powerful analytical technology to detect and quantify a large number of proteins, and is critical to understand complex biological processes and phenotypes [37, 38]. There have been a few reports of using MS strategies to identify/quantify proteins in models of embryonic development, including bovine [39], mouse [35, 40, 41], avian [42], zebrafish [43, 44], and Xenopus [45] models. While mouse/bovine are widely used model organism for studying mammalian embryo development, there are fundamental embryo and trophectodermal developmental differences in non‐human species versus humans [1, 46, 47] that dictate successful implantation [48]. Cross‐species transcriptome comparison of TE lineages at different developmental stages identified functional differences amongst non‐human versus human TEs [47]. Thus, to understand the biochemical regulatory processes, proteomics dynamics, and the kinetics of development in humans [30, 49, 50], we must ideally use human embryos and study TE lineage differentiation. This has remained technologically challenging due to the material typically required for MS‐based analysis (e.g., ∼8000 embryos required in a recent proteomic study [35]).

Development of streamlined sample preparation approaches using nanogram sample quantities with virtually lossless recovery [51] coupled with high‐resolving chromatographic system with high‐sensitivity MS analysis has enabled quantitative analysis of proteome [52]. Here, we employ this strategy to elucidate the dynamic changes of the embryo proteome during human early development from M2, 8‐cell, and blastocyst stage, and during TS differentiation into EVT and ST. We obtained an in‐depth proteome of 3974 proteins, including conserved and species‐specific markers and regulators of preimplantation embryo, embryo development, and focal adhesion/cellular/telomere maintenance. This direct proteomic analysis provides a comprehensive analysis of the dynamic protein expression in human embryos during preimplantation development and a powerful resource to enable further mechanistic studies on human trophoblast development and function.

## 2 Results

### 2.1 Proteome Profiles of Ovum, 8‐Cell Embryos and Blastocysts Are Distinct in Humans

**FIGURE 1:**
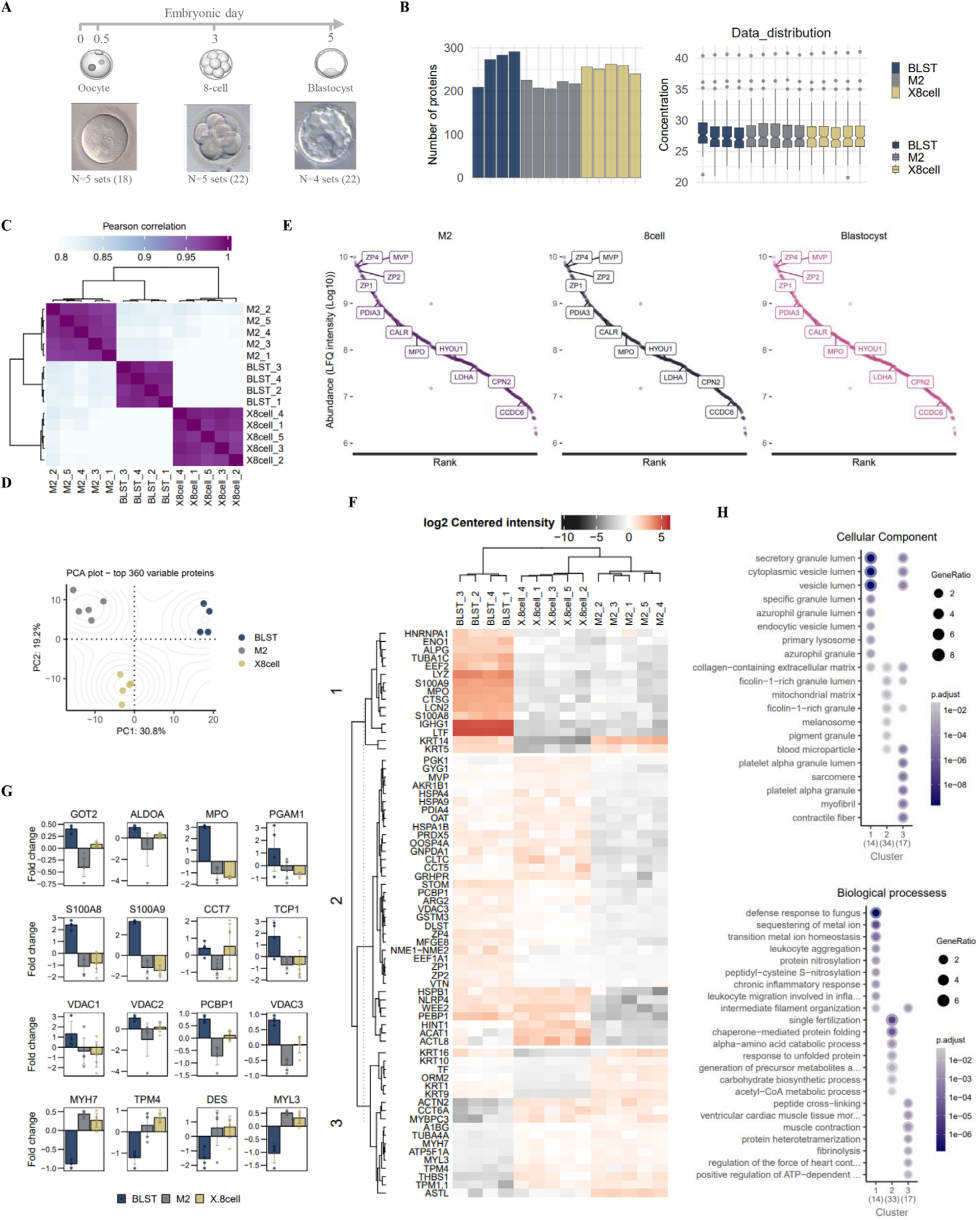
Proteome landscape of human oocyte (M2), 8‐cell embryo, and blastocysts. (A) Brightfield microscopy images of human oocyte (M2), 8‐cell embryo (X8cell), and blastocysts (BLST). (B) Number of proteins identified in replicates and their LFQ intensities across each embryonic stage (18 M2 stage samples [five sample pool], 22 8‐cell embryo samples [five sample pool], 22 blastocyst samples [four sample pool]). (C) Heatmap for Pearson correlation matrix of indicated proteomes. (D) Principal component analysis of indicated proteomes. (E) Proteins ranked based on protein abundance (LFQ intensities). Proteins implicated in preimplantation embryo development are indicated. (F) Heatmap of differentially abundant proteins (DAPs, *p* < 0.05) between proteome datasets. (G) Bar plots of indicated DAPs between the proteome datasets. (H) Dot plot showing Cellular Components and KEGG pathways enriched (*p* < 0.05) in DAP clusters identified in the proteome datasets.

Using highly sensitive sample preparation/nano liquid chromatography and quantitative MS, we first systematically monitored protein expression landscape in mature ovum (M2), 8‐cell embryos (8‐cell), and blastocysts (BLST) using data‐dependent acquisition (Figure 1A, Table S1). For sample preparation and MS analysis, 18 ovums (M2), 22 8‐cell embryos, and 22 BLST were used per sample (combined at a protein level into separate replicate analyses). Proteins were included based on stringent identification criteria; 1% peptide false discovery rate (FDR) with at least one unique peptide detected (M2: 282, 8‐cell: 321, BLST: 333 proteins detected). We restricted protein analysis to those detected in at least three replicates per stage. This filtering resulted in 212, 253, and 246 quantifiable proteins of high confidence in M2, 8‐cell, and BLST embryo stages (Figure 1B, Table S1). Unsupervised hierarchical clustering of the protein expression profiles revealed that the proteome landscape for each embryo stage was distinct (Figure 1C), which was also supported by principal component analysis (PCA), a completely different total proteome clustering method (Figure 1D). Moreover, Pearson's correlation coefficient of protein intensities between replicates in each stage is over 0.8, indicating reproducible measurement (Figure 1C). For comparative analyses of the human embryo stage proteome landscape, we employed comparative analysis with the human preimplantation embryo proteome [49, 50]. We show 164/212 proteins were co‐identified at 2‐cell stage, 175/253 proteins at 8‐cell stage, and 137/246 proteins at blastocyst stage [50], and 228/253 proteins at 8‐cell stage, and 222/246 proteins at blastocyst stage [49]. For proteins distinct in this current study (26 proteins: 8‐cell) were associated with Estrogen‐dependent gene expression, Cellular Senescence, and various signaling networks including NOTCH, WNT, and Nuclear Receptors, while for blastocyst stage (25 proteins) associated with Cytokine Signaling in Immune system and Signaling by Interleukins (Table S1).

The reported protein profiles of all stages are highly dynamic, spanning above four orders of magnitude measured by their protein abundances (LFQ intensities) (Figure 1E). Top‐ranked proteins include hallmark features of mature ovum and preimplantation embryo zona pellucida proteins [53] (ZP1/2/4) as well as known functional proteins in embryo development (PDIA3, CARL, MPO, LDHA [54]). Of the 173 commonly identified proteins across all embryo stages (M2, M8, BLST) (identified in at least three replicates per stage), we report 153 proteins and 154 proteins commonly identified at M2 stage and blastocyst stage in the human proteome based on combined single‐cell proteome analyses [49] (Table S1).

We performed protein‐wise linear model combined with empirical Bayes statistics (limma) for differential enrichment analysis to identify differentially abundant proteins (DAP) (Figure 1F) between embryo developmental stages. Using k‐means clustering, we identified distinct clusters that are regulated differently between stages (Figure 1F). Intensities (zero‐centered) for indicated proteins between stages are highlighted (Figure 1G). Higher abundance of mitochondrial proteins VDACs and GOT2 in BLST stage versus 8‐cell embryo or M2 ovum is also consistent with previous reports [55] where dense rounded or oval mitochondria are reported in M2 oocytes that remain unchanged in 16‐cell embryo until morula/blastocyst stage where it fully develops (accompanying the onset of nuclear and mitochondrial transcription [56] and mitochondrial oxygen consumption [56]). We also observed higher abundance of S100A9 in BLST stage, a hallmark feature of implantation competent blastocyst where ICM and TE display elevated expression S100A9 [57] (a secretory factor that plays a prominent role in the regulation of inflammatory processes and immune response). We further validate S100A9 in other human early embryo proteome studies at BLST stage, in addition to high abundance of VDAC (VDAC1‐3) in 2‐cell, 8‐cell, and BLAST stages [49, 50]. Keratins, which are markers of TE [58] and whose protein expression was exclusively observed in later embryonic stages in blastocyst [59], namely KRT5/14 were also found in high abundance in BLST proteome, and previous human early BLST stage embryo proteome [49].

Ontology enrichment analysis (Cellular Component, CC) revealed that proteins of high abundance in blastocyst (Cluster 1) are implicated in secretory granules (CTSG, EEF2, LCN2, LTF, LYZ, MPO, S100A8, S100A9) and mitochondria (ACAT1, ARG2, DLST, HSPA9, OAT, PRDX5) and in M2 ovum (Cluster 3) implicated in sarcomere (ACTN2, MYBPC3, MYH7, MYL3, TPM4) (Figure 1H, Table S2). This is consistent with transcriptomics studies‐based Ontology enrichment between human zygote and blastocysts (trophectoderm) (Figure S1).

Biological processes enriched in later stages (Cluster 1 and 2) include defense response (CTSG, LTF, MPO, S100A8, S100A9) and leukocyte aggregation (S100A8, S100A9) for immune regulation during implantation [60], and regulation of apoptotic signaling pathway (ENO1, S100A8, S100A9) for blastocoel formation [61], chaperone‐mediated protein folding (CCT5, HSPA1B, HSPA9, HSPB1, PDIA4) to ensure proper folding of functional proteins for preimplantation embryo development [62], carbohydrate metabolism (AKR1B1, CLTC, GYG1, PGK1) essential to blastocyst development [63] (Figure 1H, Table S2).

### 2.2 Stage‐Specific Differentially Abundant Proteins in Preimplantation Embryo Highlight Protein Players of Hallmark Process of Preimplantation Embryo Development

**FIGURE 2:**
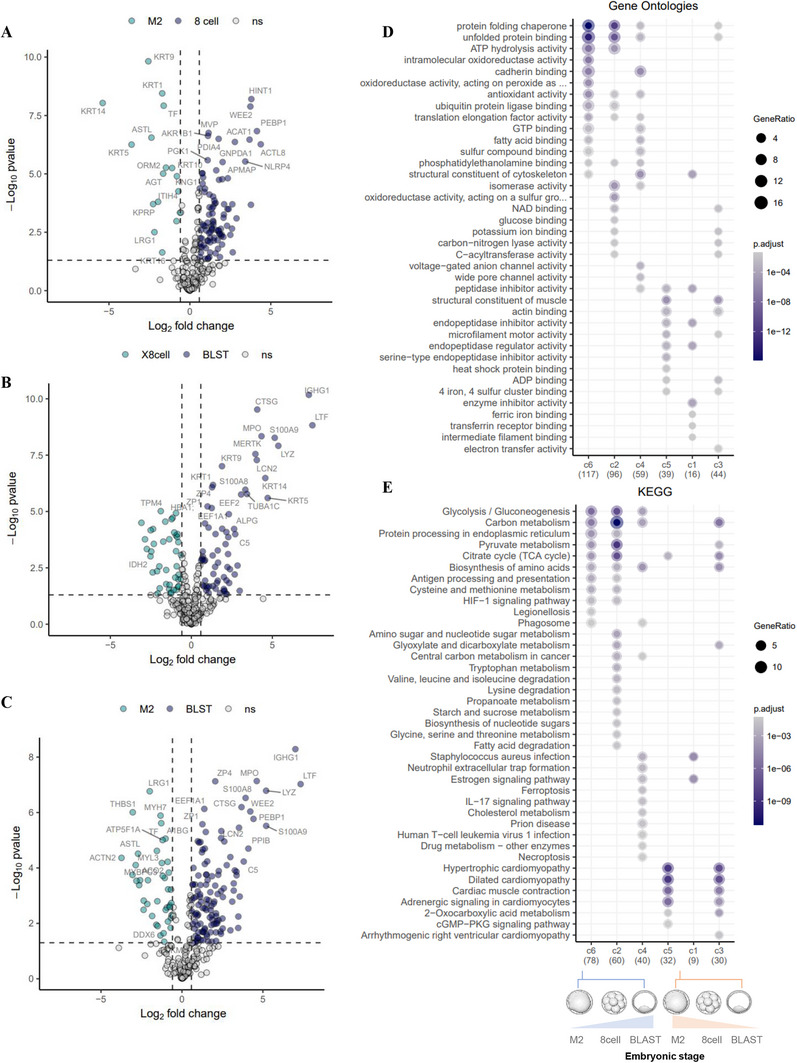
Differentially abundant proteins after pairwise comparisons of human oocyte (M2), 8‐cell embryo, and blastocysts. Volcano plots showing differentially abundant proteins (*p* < 0.05, FC > 1.5) in (A) 8 cell versus M2, (B) blastocyst versus 8 cell, and (C) blastocyst versus M2, and their enrichment for (D) Biological Processes and (E) KEGG pathways.

Next, we performed pairwise comparisons of human oocyte (M2), 8‐cell embryo, and blastocysts, to interrogate stage‐specific DAPs (8 cell vs. M2, BLST vs. 8 cell, BLST vs. M2) (Figure 2A–C, Tables S4–S6). We also performed Gene Ontology (CCs and Biological Process, BP) and KEGG pathway enrichment to identify processes/pathways temporally regulated (Figure 2D,E). Significantly DAPs (*p* < 0.05) include 97 up‐ and 17 down‐regulated proteins between 8 cell versus M2, 65 up‐ and 45 down‐regulated proteins between BLST versus 8 cell, and 123 up‐ and 41 down‐regulated proteins between BLST versus M2 (Tables S4–S6). Importantly, we highlight 33 proteins previously identified as differentially regulated between BLST versus 8 cell [49] commonly downregulated in expression (e.g., PKM, important in regulating pyruvate kinase activity in embryo), and 15 proteins commonly enriched [49] in expression (e.g., NCL – transcriptional regulator of EGFR and LIF signaling, and PRDX1 – regulates intracellular levels of reactive species through signaling cascades of growth factors) (Table S5). Proteins uniquely identified associated with BLAST versus 8‐cell stage (18/110) in this current study were associated with Long‐term potentiation, Neurotransmitter receptors and postsynaptic signal transmission, Syndecan interactions, and Cell recruitment (pro‐inflammatory response).

Overall, upregulated proteins in BLST versus M2 were implicated in CCs such as secretory granules and chaperonin‐containing T complex, and BPs such as protein folding and stabilization, telomerase RNA localization, DNA biosynthesis, cellular detoxification, and chromosome organization (Table S7). To identify specific KEGG pathways enriched in each cluster across embryonic stage, we performed pairwise comparison between M2, 8‐cell embryos, and blastocysts (Figure 2D,E, Tables S10–S12). Differential clusters include glycolysis, amino acid biosynthesis, and antigen processing/presentation (high in M2/8‐cell stage) and metabolite processing (8‐cell stage). In contrast, lower‐abundant proteins in BLST implicated in sarcomere, contractile fibers, and myofibril localization, and BPs such as muscle contraction and cardiomyocyte signaling (Figure 2E, Tables S7,S11,S12).

#### 2.2.1 8‐Cell Embryos versus M2

High abundant proteins in 8 cell versus M2 were implicated in CCs such as chaperonin‐containing T complex, mitochondrial matrix, granules, and focal adhesion, and BPs such as protein folding, establishment of protein localization, generation of precursor metabolites, and telomere maintenance (Figure 2A,D,E, Table S8). KEGG pathways that were enriched include carbon metabolism (pyruvate, glycolysis, TCA cycle), biosynthesis of amino acids, and protein processing in ER (Tables S8,S12).

#### 2.2.2 Blastocysts versus 8‐Cell Embryos

Upregulated proteins in blastocysts versus 8‐cell embryos were implicated in CCs such as secretory granules, pore complex, and collagen‐containing ECM, and BPSs such as intermediate filament cytoskeleton, defense response, glycolytic process, ATP generation, metal ion sequestration, telomere maintenance, and leukocyte maintenance. KEGG pathways that were enriched include Glycolysis, Neutrophil extracellular traps, biosynthesis of amino acids, ferroptosis, and IL‐17 signaling (Figure 2B,D,E, Table S9). KEGG pathways that were enriched include carbon metabolism (Glycolysis, pyruvate metabolism), biosynthesis of amino acids, protein processing in ER, ferroptosis, and necroptosis (Tables S9,S12).

#### 2.2.3 Correlation of Proteome and Transcriptome of Embryo Developmental Stage

**FIGURE 3:**
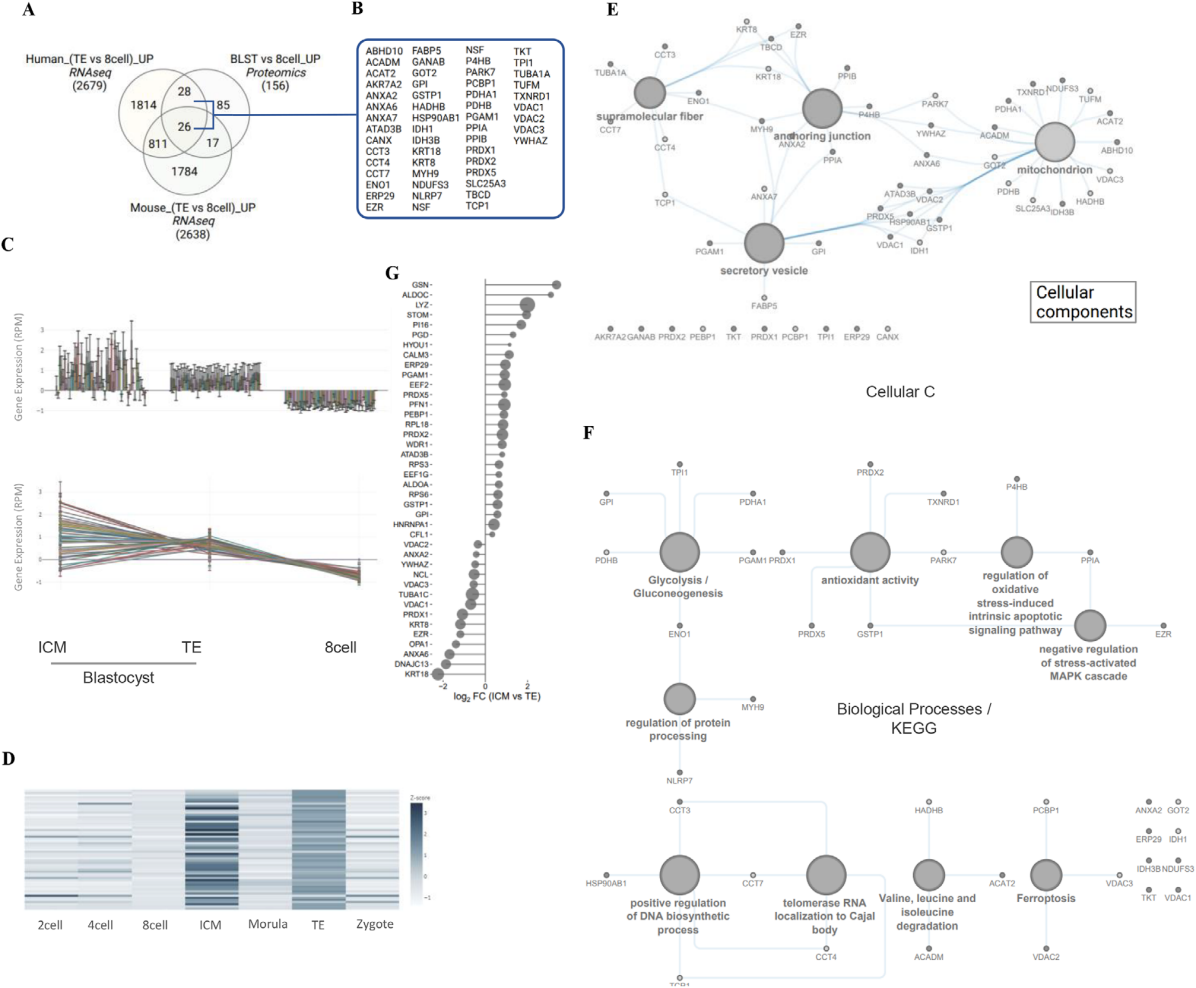
Comparative multi‐omic analysis and proteome network analysis of DAPs in blastocysts versus 8‐cell embryos. (A) Venn diagram of RNAseq transcriptomics‐based transcripts enriched in blastocyst versus 8‐cell embryo (human or mouse) [64] with high abundant proteins in blastocyst versus 8‐cell embryo in our proteome. (B) Proteins upregulated in blastocyst versus 8‐cell embryo, in addition to transcript level; referred to as BLST proteins. RNAseq transcriptomics based normalized transcript levels of BLST proteins [64] (C) normalized transcript levels 8‐cell embryo, and blastocyst (TE or ICM) as well as (D) in all developmental stages of preimplantation embryos. Network analysis of BLST proteins for (E) Cellular Components and (F) Biological Processes/KEGG pathways and associated proteins. (G) Differential abundance of transcript levels of BLST proteins in ICM versus TE.

For comparative analyses of the human embryo stage proteome landscape, we employed comparative analysis with a multi‐omics database of human and mouse early embryo [64]. Of the 156 significantly higher abundant proteins in BLST versus 8‐cell embryos, we verified RNAseq‐based upregulated expression (transcript levels) of 71 proteins in human or mouse blastocysts (trophectoderm, TE or ICM) compared to 8‐cell embryos (Figure 3A,B). Whereas transcript levels of 26 proteins were consistently upregulated in human and mouse, 28 were uniquely upregulated in humans (Figure 3C). Interestingly, transcripts levels of these 54 proteins (26 + 28) reflect temporally upregulated expression throughout the preimplantation stages of human embryos, from zygote, 2 cell, 4 cell, 8 cell, morula, and blastocyst stage (TE & ICM) (Figure 3D).

We surmise that proteins in different pathways are implicated as networks linking proteins and biological concepts (Figure 2D,E, Tables S10–S12). These proteins were implicated in CCs such as secretory granules/vesicles, mitochondria, anchoring junctions, and supramolecular fiber, and BP/KEGG pathways such as glycolysis, antioxidant activity, regulation of apoptotic signaling pathway, DNA biosynthesis, telomerase maintenance, and ferroptosis. These processes are well‐studied for their contribution in preimplantation embryo development. Furthermore, we highlight the differential abundance of transcripts for these 58 proteins in ICM versus TE in human blastocysts. Higher abundance of glycolytic proteins and mitochondrial proteins between ICM and TE, respectively, further support differential metabolic needs between these lineages [65] (Figure 3G).

Thus, our data provides a context to understand the dynamic events during early embryo development in humans at a proteome level and correlation at a transcript level, but can also identify key regulatory factors that modulate these events.

### 2.3 Proteomic Analysis of Trophoblast Lineages Arising from the Trophectoderm Cells of the Human Blastocyst

**FIGURE 4:**
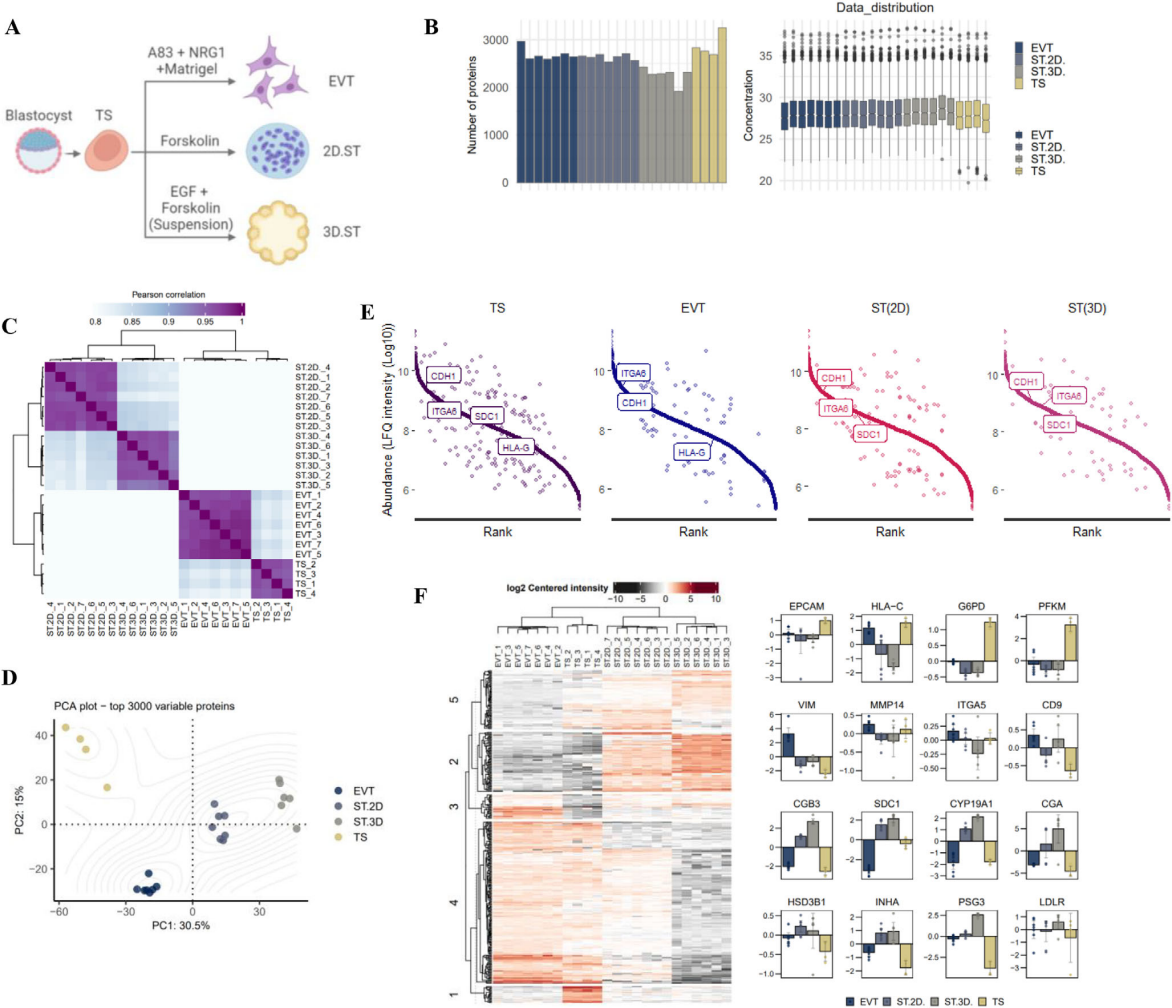
Proteome landscape of trophoblast stem cells (TSblast) and differentiation into EVTs and STs. (A) Differentiation of trophoblast stem cells (TSblast) into extravillous trophoblasts (EVTs) and syncytiotrophoblasts (STs). (B) Number of proteins identified in biological replicates and their LFQ intensities for TS (*n* = 4), EVT (*n* = 7), and STs differentiated into 2D or 3D cell models; ST(2D)‐TS^blast^ (*n* = 7) and ST(3D)‐TS^blast^ (*n* = 6), respectively. (C) Heatmap for Pearson correlation matrix of indicated proteomes. (D) Principal component analysis of indicated proteomes. (E) Proteins ranked based on protein abundance (LFQ intensities). Proteins implicated in preimplantation embryo development are indicated. (F) Heatmap of differentially abundant proteins (DAPs, *p* < 0.05) between proteome datasets. (G) Bar plots of indicated DAPs between the TS and differentiated proteome datasets.

Following attachment of the hatched blastocyst (via trophectoderm), the trophoblast cells differentiate into extravillous trophoblasts (EVTs) that facilitates embryo invasion into endometrium for intrauterine embryo development, and ST that form the placenta [13, 15, 16, 17]. We next interrogated how the protein landscape of trophoblast stem (TS) cells directly derived from human blastocysts change during their differentiation into EVT or STs (Figure 4A). As reported by our team previously, TS cells (TSblast) have been demonstrated to differentiate into EVT‐ and ST‐like cells [17] at a 2D (designated ST(2D)) and 3D culture level (designated ST(3D)) [17].

We identified a total of 3814, 3710, 3574, and 3169 proteins in TSblast, EVT, ST(2D), and ST(3D), respectively (Figure 4B, Table S13), with 105 proteins co‐identified in all studies independent of lineage associated with cell adhesion/cadherin binding (MACF1, CLIC1), mRNA binding (HSP90AA1, HNRNPC, PKM, DDX5, HNRNPA2B1), regulation of biological quality (CF1, SLC9A3R1, DIAPH1, CTNND1, LMNA, ANXA1, KRT1), and developmental process (anatomical structure; DIAPH1, CTNND1, FLNB, PGK1, KRT14, VDAC1, TGM2, CALR, KIF5B, tissue development; FASN, ACTG1, TAGLN, TJP1, TCOF1, PLEC).

Various human trophoblast cell markers have been described [17, 66], with their expression identified in various trophoblast lineages (KRT7 – identified in all TS lineages, and TFAPC2 – identified in TSblast and ST(3D) proteome), HLA class I profile (identified in TSblast, EVT and ST(2D) proteome). We also did not identify NCAM1, previously shown poorly expressed in primary EVT and EVT‐like cells [17].

From our previous studies, we concur that TSblast cells are human TS cells, in addition to their differentiated lineages. Unsupervised hierarchical clustering of the protein expression profiles revealed that the protein profiles were distinct (Figure 4C), which was also supported by PCA (Figure 4D), with correlation in proteomes of ST(2D) and ST(3D). The reported proteomes are highly dynamic, spanning about four orders of magnitudes (Figure 4E) with mutually exclusive detection of EVT marker HLA‐G [17] and ST marker SDC1 [17] between EVT and ST(2D/3D) proteomes, respectively.

DAP analysis revealed previously reported molecular features that are lineage‐specific (Figure 4E,F, Table S14). Epithelial to mesenchymal transition of TS to EVT is evident at a molecular level from down regulation of EPCAM and upregulation of VIM, matrix degradation proteins MMP14 and invasion‐related proteins ITGA5 and CD9 (Figure 4F). On the other hand, proteins implicated in ST formation including CGB3, SDC1 and CGA were upregulated in ST(2D) and ST(3D) proteomes (Figure 4F).

Previous report [67], including ours [17], have demonstrated that 3D culture enhances differentiation of choriocarcinoma cells into ST‐like cells. Consistent with our previous report, our proteomic profiling also identified higher abundance of ST markers CGB and SDC1 in ST(3D) versus ST(2D). Thus, for downstream analysis, we only include ST(3D) proteome for comparative analyses.

**FIGURE 5:**
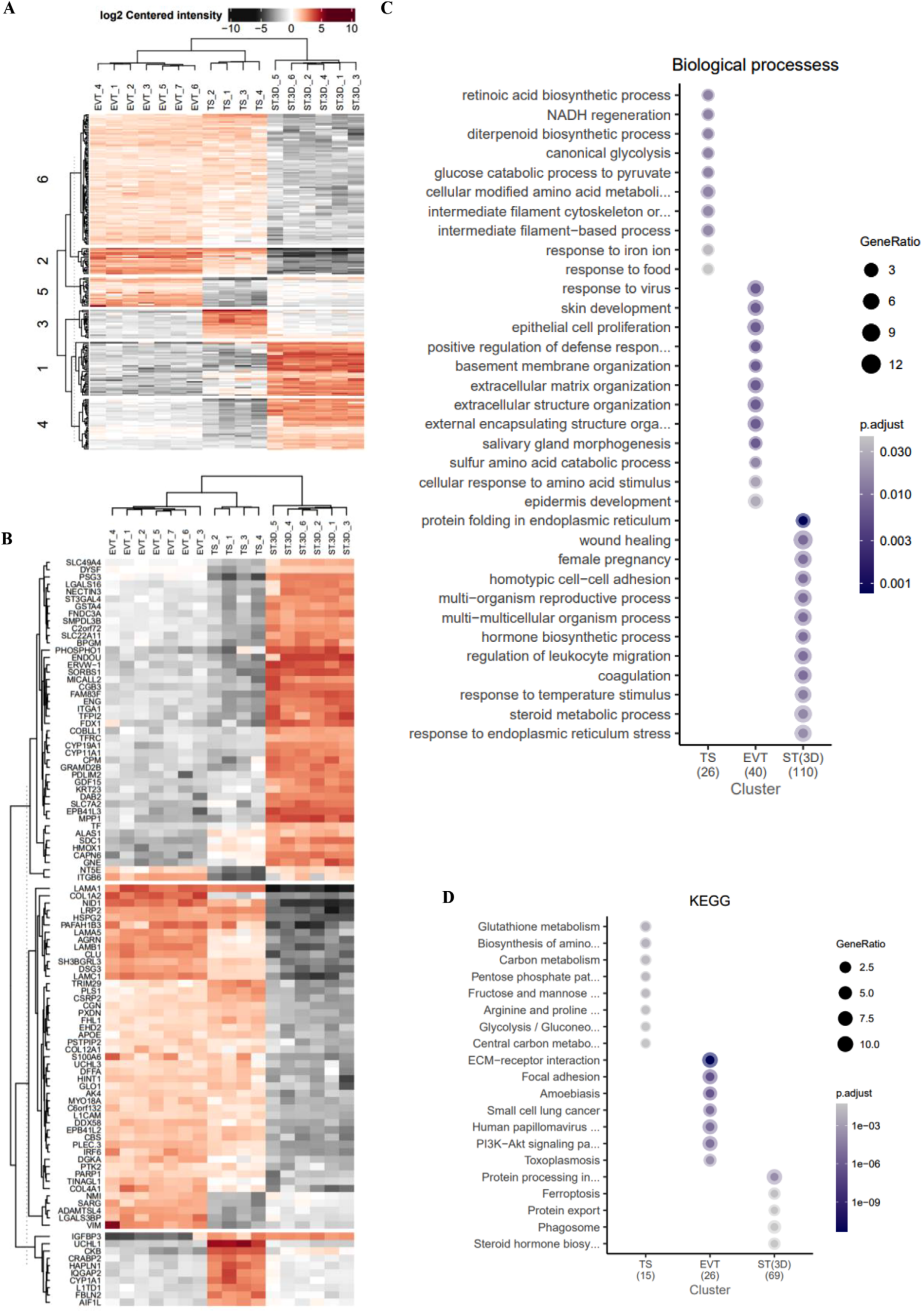
Hierarchical clustering of differentially regulated processes during TS differentiation into EVTs and ST(3D). (A) Heatmap of DAPs (*p* < 0.05) in TS, EVT, and ST(3D) proteomes. (B) Heatmap of DAPs (*p* < 0.005, FC > 3) in TS, EVT, and ST(3D) proteomes. Enrichment analysis of DAPs for (C) Biological processes and (D) KEGG pathways.

DAP analysis (*p* < 0.05, FC > 1.5) of TS, EVT, and ST(3D) proteomes identified lineage‐specific enriched protein clusters; 27, 43, and 119 proteins in TS, EVT, and ST(3D), respectively (Figure 5A, Tables S14,S15). We further highlight protein clusters with a more stringent cut‐off (*p* < 0.005, FC > 5) and identity potential novel markers of these three sub‐populations of trophoblasts (Figure 5B, Table S14). These include syncitiotrophoblast marker SDC1 and CYP19A1 in ST(3D) and EMT marker VIM, SARG, NMI, ADAMTSL4, and LGALS3BP in EVT lineages.

For comparative analyses of each TS‐derived lineage, we performed analyses relative to the human Tsc proteome [30]. We report 26/27 (TS), 37/43 (EVT), and 109/119 proteins in the human Tsc proteome. Ranked‐based abundance relative to Tsc proteome, highlighted 15 proteins in TS more abundant in human protein expression than other cell types (EVT, ST(3D)); significantly enriched networks included Formation of the cornified envelope, KEAP1‐NFE2L2 pathway, Developmental Cell Lineages (KRT80, KRT17), Glucose metabolism (ENO2, PFKM), and Metabolism of carbohydrates and carbohydrate derivatives (G6PD, ENO2, PFKM) (Table S14).

We performed enrichment analysis for BPs and KEGG pathways in these protein clusters (Figure 5C,D, Table S15). While TS protein clusters displayed striking enrichment for metabolic processes (BPs: glycolysis, NADH regeneration, retinoic acid biosynthesis and KEGG: amino acids, PPP pathway, glycolysis), EVT protein clusters were enriched for invasion related processes (BPs: ECM organization, basement membrane organization and KEGG: ECM receptor interaction, focal adhesion, PIS‐AKT signaling). In contrast, ST(3D) protein clusters were implicated in secretory function (BP: protein processing and folding in ER, steroid metabolism, and KEGG: protein export) and coagulation.

Protein cluster enriched specifically in EVTs (compared to TS and ST(3D)) were implicated in ECM organization (ADAMTSL4, COL1A2, COL4A1, LAMB1, LAMC1, NID1) via receptors (FBN2, ITGB6, VIM, COL1A2, LRP2) (Table S15). In contrast, Protein cluster enriched specifically in ST(3D) were implicated in protein folding in endoplasmic reticulum (CALR, CANX, DNAJC3, PDIA3), female pregnancy (ENDOU, HSD11B2, PSG3, SLC38A2, ACSL4, CALR, SLC2A1, STS), hormone biosynthetic process (CYP11A1, CYP19A1, DAB2, FDX1, POR), homotypic cell‐cell adhesion (CSRP1, FN1, HSPB1, LYN, PLPP3, STXBP1), regulation of leukocyte migration (CYP19A1, HMOX1, MPP1, ST3GAL4, CALR, DPP4, LGMN, LYN) and coagulation (ST3GAL4, TFPI, TFPI2, CSRP1, FN1, HSPB1, LYN, STXBP1).

### 2.4 Differentially Abundant Proteins After Pair Wise Comparisons Between TS, EVTs, and ST(3D)

**FIGURE 6:**
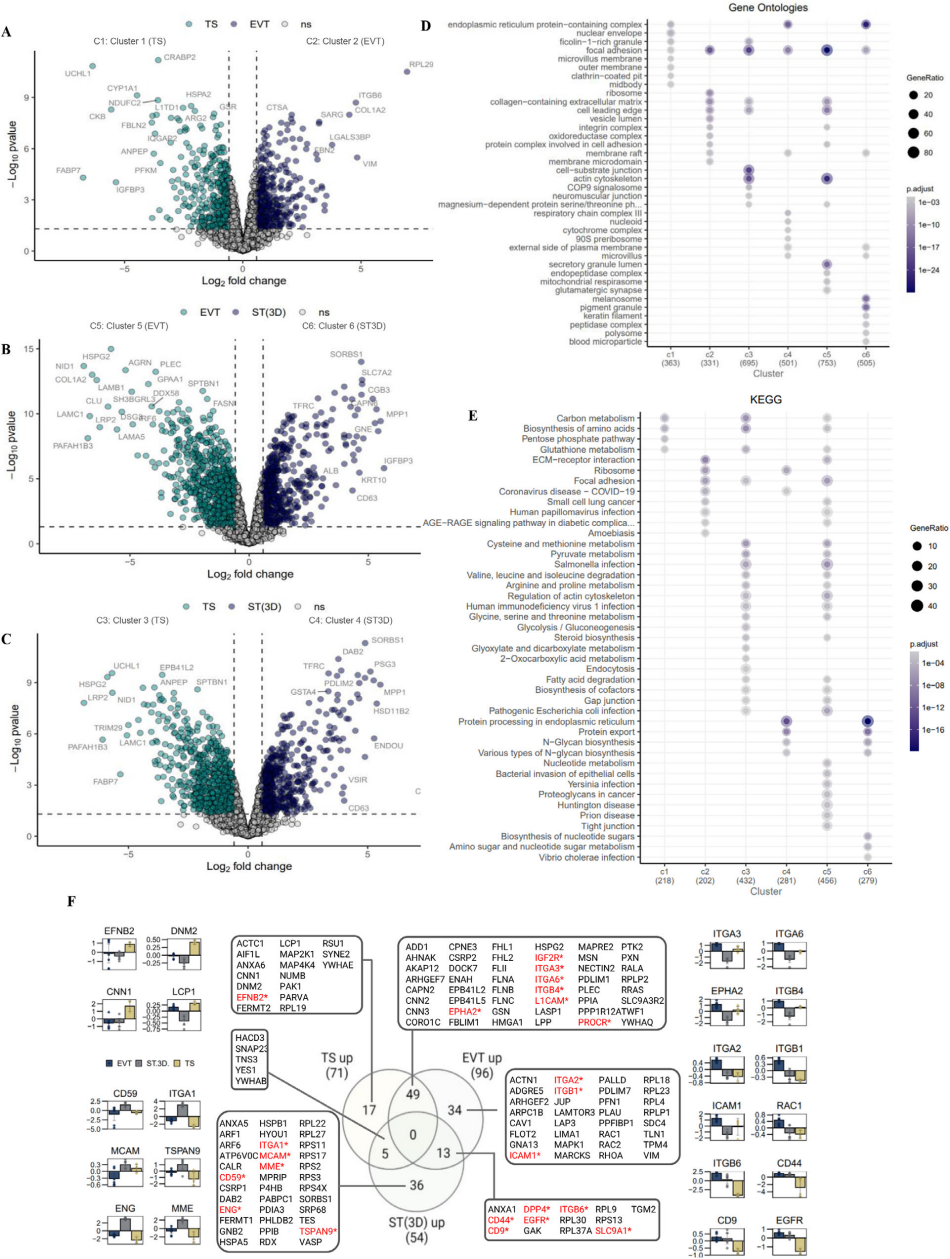
Differentially abundant proteins after pair wise comparisons between TS, EVTs, and ST(3D). Volcano plot of DAPs (*p* < 0.05, FC > 1.5) in (A) EVT versus TS, (B) ST(3D) versus TS, and (C) ST(3D) versus EVT proteomes. (D) Gene Ontology (Cellular Components) enrichment in DAPs after pair wise comparisons between TS and differentiated cell lineages. (E) KEGG pathways enriched in DAPs after pair wise comparisons between TS and differentiated cell lineages. (F) Proteins associated with “focal adhesion” KEGG pathway term are enriched in DAPs after pairwise comparisons between TS and its lineages. Corresponding bar plots with relative protein abundance (based on LFQ intensities) between different TS lineages for indicated proteins. Proteins in red* are annotated as cell surface proteins by Surfaceome predictor SURFY [69].

We next performed pairwise comparisons to interrogate DAPs during TS differentiation to either EVT or ST(3D). We also performed pairwise comparisons between two lineages, ST(3D) and EVT, to identify their hallmark processes (Figure 6A–C, Tables S16–S18). We identified a total of 704 and 1209 DAPs (*p* < 0.05) during TS differentiation to EVTs and STs, respectively. We observed a larger proteome‐subset of 1274 DAPs between ST(3D) versus EVTs, with 510 and 764 proteins with significantly higher abundance in ST(3D) and EVTs, respectively.

We surmise these DAPs following pairwise comparisons for each lineage in Table S19 and present their enrichment analysis results in Figure 6D,E (Table S20).

#### 2.4.1 TS differentiation to EVTs (Figure 6A)

During TS differentiation to EVTs, 341/704 proteins were found in higher abundance in EVTs versus TS, including a variety of integrins (ITGA2, ITGA3, ITGA6, ITGB1, ITGB4, ITGB6), COL1A2, CTSA, LAMB1, and EGFR implicated in invasion through ECM. CCs/BPs/KEGG pathways enriched in upregulated proteins include focal adhesion, cell‐substrate junction, and cell leading edge, which are implicated in cell‐substrate adhesion, EMC‐receptor interaction, and migration, potentially via an integrin‐mediated signaling pathway. On the other hand, 363/704 proteins displayed lower abundance and were enriched in glycolysis and metabolite transporters (SLC19A3, SLC1A3, SLC25A4, SLC27A2, SLC27A6, SLC2A3, SLC7A2, SLC7A5, SLC7A8, SMCHD1).

#### 2.4.2 TS differentiation to ST(3D) (Figure 6C)

During TS differentiation to ST(3D), 510/1209 proteins were found in higher abundance in ST(3D) versus TS and were endoplasmic reticulum‐Golgi proteins implicated in N‐Glycan biosynthesis. Glycosylation of proteins determines many of their final properties, thus becoming essential for the embryo‐maternal relation during implantation and placentation. Other pathways enriched include ribosomes/translation preinitiation complex, protein export, and biosynthesis processes such as amino acids, nucleotides, sugars). On the other hand, 699/1209 proteins displayed lower abundance and were implicated in glycolysis.

#### 2.4.3 EVT versus ST(3D) proteomes (Figure 6B)

DAPs in EVTs and ST(3D) proteomes highlight processes that are lineage specific. Indeed, of 1274 DAPs between ST(3D) versus EVTs, 764 proteins that are of higher abundance in EVTs were enriched for invasive function (focal adhesion, actin filament organization, integrin signaling and ECM interaction) whereas 510 proteins that are of higher abundance in ST(3D) were enriched for secretory functions (ER‐Golgi proteins, protein export, steroid biosynthesis).

#### 2.4.4 Functional Insights in Focal Adhesions

As an example, we highlight here how can gain molecular insights into reassignment of proteins implicated in focal adhesion (Figure 6F, Table S21) occurs between TS and their lineages, in particular the integrin switch important for coordinating different subpopulation invasive phenotypes [68]. A total of 71, 96, and ST(3D) focal adhesion proteins were found in higher abundance in each sub‐population compared to one or both subpopulations. Interestingly, we show 16/17 TS proteins associated with Tsc human proteome [30], with unique cytoskeletal actin ACTC1 abundantly linked with fetal development of skeletal and cardiac muscle. We highlight proteins (in red*) that are annotated as surface proteins based on SURFY [69]. During TS differentiation to EVTs, we observe elevated expression of ITGA3, ITGA6, and ITB4, but their expression is downregulated during TS differentiation towards ST(3D). In contrast, ITGA2 and ITGB1 displayed higher abundance in EVTs alone. Compared to TS, both EVT and ST(3D) displayed higher abundance of ITGB6, albeit to greater levels in EVTs. Tetraspanins like CD44 and CD9 were found in higher abundance in EVTs and ST(3D) at similar levels. In contrast, ITGA1, CD59, and ENG were found in higher abundance exclusively in ST(3D).

#### 2.4.5 Comparing Proteomics with Transcriptomics Data

We verified differential expression of DAPs in ST(3D) versus EVTs with RNAseq‐based transcript levels in ST and EVTs derived from human placenta or established from blastocysts (published in our previous study [17]) (Table S22). Specifically, transcript levels of 254/482 high abundant proteins in ST(3D) versus EVTs were also upregulated in ST(3D) versus EVT cell lines established from human blastocysts. Moreover, transcript levels of 192/482 of these proteins were also upregulated in primary ST versus primary EVTs (isolated from human placental tissues). In contrast, transcript levels of 575/764 high abundant proteins in EVTs versus ST(3D) were also upregulated in EVT versus ST(3D) established from blastocysts. Furthermore, transcript levels of 643/726 of these proteins were similarly upregulated in primary EVT versus primary ST transcriptome data.

**FIGURE 7:**
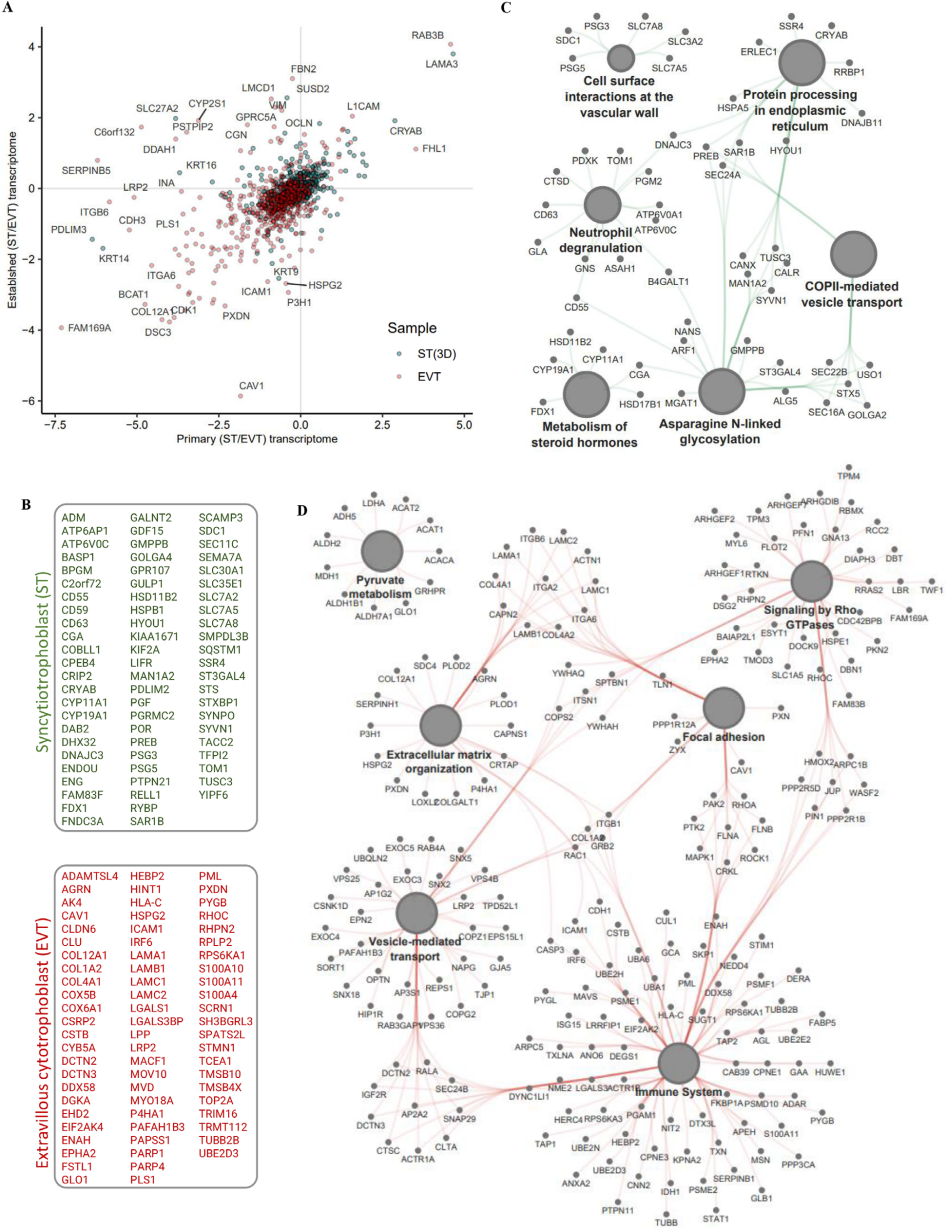
Comparative proteomic and transcriptomic analyses uncover EVT and ST function. (A) Correlation of RNAseq‐based transcript abundance of DAPs (ST(3D) vs. EVT) between primary and blastocyst‐derived ST versus EVTs from our previous study [17]. Proteins that are of higher abundance in ST(3D), regardless of their transcript abundance, are coded blue. Proteins that are of higher abundance in EVTs, regardless of their transcript abundance, are coded red. (B) Correlation analysis of the top 70 proteins whose protein and transcript abundance correlated in ST or EVT are indicated. (C) Protein network map of proteins and KEGG/Reactome pathways enriched in proteins whose protein and transcript abundance were higher in ST compared to EVTs, while (D) indicates protein network map of proteins/KEGG/Reactome pathways whose protein and transcript abundance were higher in EVTs compared to ST.

We highlight those proteins that are similarly dysregulated in transcriptome data and our proteome data (Figure 7A,B, Table S22), which includes 537 and 136 in EVT and ST(3D), respectively. We constructed a protein network indicating linkages of proteins and biological concepts (KEGG pathways) (Figure 7C,D). Pathways enriched in ST(3D) proteins were implicated in N‐linked glycosylation, COPII‐mediated vesicle transport, neutrophil degranulation, and cell surface interaction at the vascular wall. EVT proteins were implicated in Rho GTPases, focal adhesion, ECM organization, and immune regulation.

Thus, our data not only provide biological processes and pathways regulated during TS lineage differentiation but also uncover potential protein drivers.

## 3 Discussion

**FIGURE 8:**
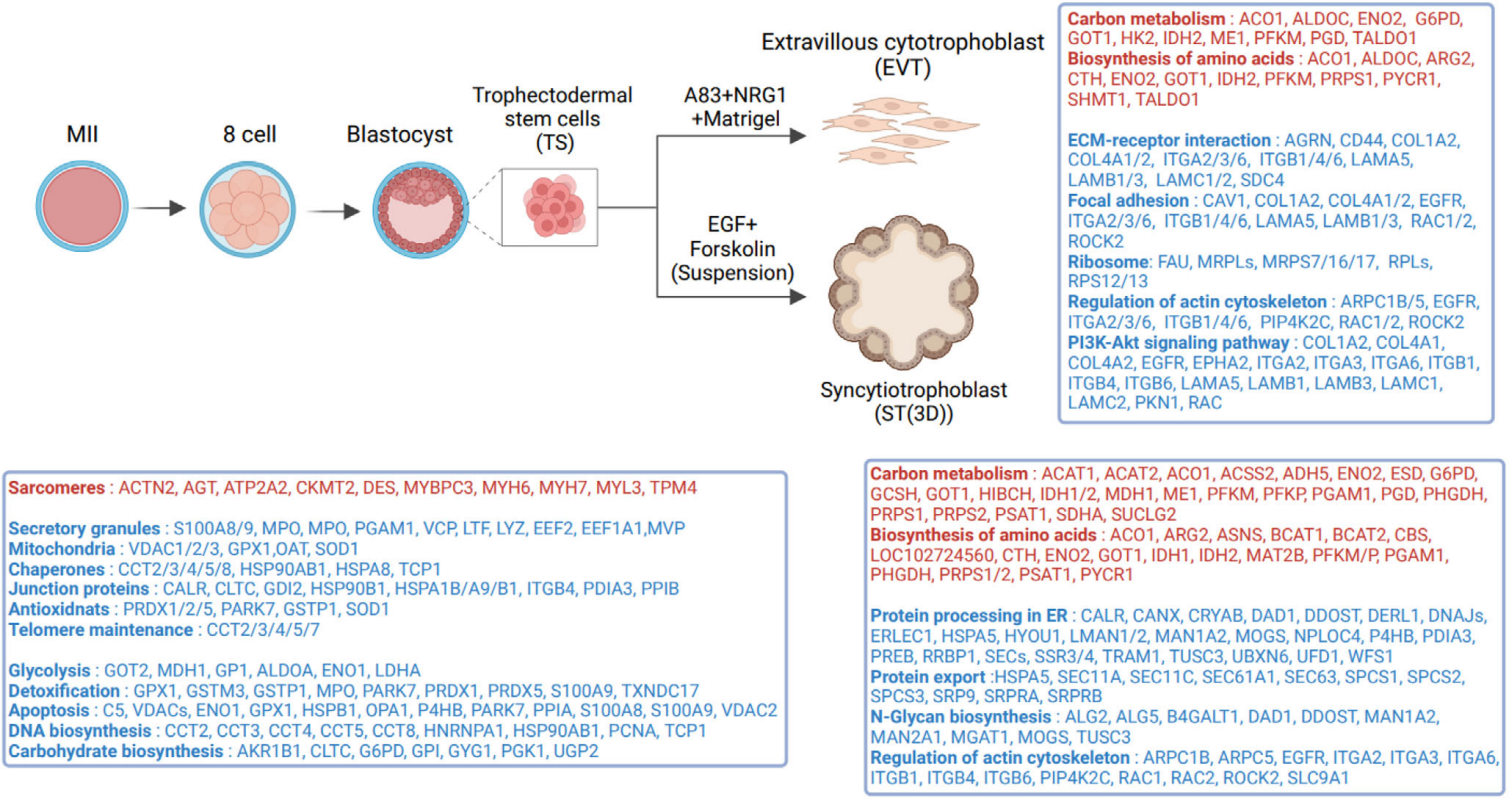
Summary of protein landscape reprogramming and associated processes during early human embryogenesis and trophoblast function.

Herein, using quantitative MS‐based proteomics, we systematically monitored the protein expression landscape and their dynamic regulation between human ovum (M2), 8‐cell embryo, and blastocysts stage, and blastocyst lineage‐specific differentiation into EVTs and differentiated ST, highlighting biological processes that are temporally regulated in humans in preimplantation embryos and blastocyst‐lineage specification. This study provides a comprehensive landscape of proteome dynamics of human embryo (preimplantation) development and trophoblast differentiation, and direct insight into the molecular features of the developmental process. Herein, we obtained an in‐depth proteome of 3974 proteins, including conserved and species‐specific markers and regulators of preimplantation embryo, embryo development, and focal adhesion/cellular/telomere maintenance. These findings extend our limited knowledge of the sequential order of protein landscape reprogramming and associated processes during early human embryogenesis and trophoblast function (Figure 8).

A major challenge to this field has been to identify protein regulators that participate in preimplantation development in humans due to ethical consideration and paucity in material required [49]. Technical advances in sample preparation amenable for MS have enabled the proteome study of complex systems with limited material. Development of streamlined sample preparation approaches using (sub)nanogram sample quantities [49, 50] with virtually lossless recovery [51] coupled with a high‐resolving chromatographic system with rapid acquisition MS analysis has enabled quantitative analysis of proteome [52]. With recent advances in isolation strategies combined with MS‐based proteomics of single (embryo) cells [49, 50], and applications of transcriptome and epigenome has significantly expanded our understanding of the dynamics of early embryo development [70, 71, 72, 73, 74, 75], mRNA abundance is not always direct proxy to protein abundance, localization and hence function [76]. Here, we provide evidence showing dynamic expression of proteins found in secretory granules, mitochondrial proteins, junction proteins, chaperones, and antioxidants. Comparative analysis between previously reported transcriptomic and proteomic data also disclosed several of these processes, namely glycolysis, mitochondria, antioxidant activity, and anchoring junctions. Furthermore, we highlight the differential abundance of transcripts for these proteins in ICM versus TE in human blastocysts. Higher abundance of glycolytic proteins and mitochondrial protein expression between ICM and TE further support differential metabolic needs between these lineages [65].

Similarly, conserved functions during TE lineage specifications are revealed, co‐identified in all studies, independent of lineage, associated with various cell communication/surface/cell adhesion function, and development/tissue/cell organization processes. Further, through comparative analyses of human Tsc proteome, and pre‐ and post‐implantation stem cell models we highlight unique key metabolic regulators in our proteome of TS, EVT, and STs associated with developmental cell lineages [30]. Placental hormones secreted by ST are required for the establishment and maintenance of pregnancy, adaptation of the maternal organism to pregnancy, and fetal growth [77]. N‐glyco of protein is important in fusion to form ST [78]. STs, are fetal‐derived cells that lie at the maternal‐fetal interface and tightly regulate the exchange of nutrients, metabolites, and other macromolecules between these two entities. Indeed, we see various glucose transporters (SLC38A2, SLC7A2, SLC22A11, SLC38A9, SLC49A4, SLC9A1, SLC7A8, SLC2A1).

Overall, our data not only provide a valuable resource for further mechanistic studies of select proteins in preimplantation embryos, but also suggest additional players and regulatory mechanisms governing early embryo development. Further, we provide new insights into understanding trophoblast differentiation and lineage at a proteome level, with correlation with their transcriptome, and application in human trophoblast development and function.

## 4 Experimental Procedures

### 4.1 Human Oocytes and Morula Embryo Collection

All donated oocytes and embryos were collected after the consent and approval of the patients based on the guidelines of the ethical committee approved by the Royan Institute Human Research Ethics Committee (Ref: IR.ACECR.ROYAN.REC.1395.8), and all experiments were performed in accordance with protocol guidelines and regulations. All donors were informed that their oocytes or embryos would be used for basic research to understand the mechanism of in vitro oocyte maturation and that the donation would not affect their clinical treatment. Patients who underwent assisted reproductive technology (ART) treatment between 2017 and 2019 were enrolled in the study. From E4‐E7, embryos were cultured under standard conditions. Microscopes (magnification of ×200 to ×400) were used to assess the integrity and maturity of the denuded oocytes. Embryos were fixed and stained as previously described [79]. Embryos were mounted in PBS and placed between two cover glasses using silicon spacers (Grace Bio‐labs) prior to morphological assessment. All embryos developed to blastocysts, with a distinct ICM (epiblast [EPI] and primitive endoderm (PE). Measurements we made to assess the number of cells per lineage and blastocyst diameter. All human MII oocytes, M8 cell stage, and blastocyst embryos were collected from donors using a standard protocol. To minimize the individual heterogeneity resulting from individual donors and handling time, we obtained oocyte (18 ovums, pooled into five sets), 8 cell (22 8‐cell stage embryo's, pooled into five sets), and blastocyst stage (22 blastocysts, pooled into four sets). The zona pellucida of each embryo was not removed (we did not employ Tyrode's solution, acidic solution, or any washing stages) prior to proteomic analysis. Samples from individual donors were pooled to overcome limitations in protein/peptide yield for proteome analysis.

### 4.2 Culture and Differentiation of Human Trophectoderm Cells

Trophectomderm stem cells (TS) were obtained as a gift from Hiroaki Okae and cultured as described previously [17] in TS medium [DMEM/F12 supplemented with 0.1 mM 2‐mercaptoethanol, 0.2% FBS, 0.5% Penicillin‐Streptomycin, 0.3% BSA, 1% ITS‐X supplement, 1.5 µg/mL L‐ascorbic acid, 50 ng/mL EGF, 2 µM CHIR99021, 0.5 µM A83‐01, 1 µM SB431542, 0.8 mM VPA, and 5 µM Y27632]. Cells were cultured at 37°C in 5% CO2 and the culture medium was replaced every 2 days.

TS^blast^ cells were seeded in a 6‐well plate pre‐coated with 1 µg/mL Col IV at a density of 0.75 × 10^5^ cells per well and cultured in 2 mL of EVT medium [DMEM/F12 supplemented with 0.1 mM 2‐mercaptoethanol, 0.5% Penicillin‐Streptomycin, 0.3% BSA, 1% ITS‐X supplement, 100 ng/mL NRG1, 7.5 µM A83‐01, 2.5 µM Y27632, and 4% KnockOut Serum Replacement]. Matrigel was added to a final concentration of 2% shortly after suspending the cells in the medium. At Day 3, the medium was replaced with the EVT medium without NRG1, and Matrigel was added to a final concentration of 0.5%. After the cells reached ∼80% confluence at Day 6, they were dissociated with TrypLE for 15–20 min at 37°C and passaged to a new Col IV‐coated 6‐well plate at a 1:2 split ratio. The cells were suspended in the EVT medium without NRG1 and KSR, Matrigel was added to a final concentration of 0.5%, and cultured for two additional days.

For the induction of ST(2D)‐TSblast cells, TSblast cells were seeded in a 6‐well plate pre‐coated with 2.5 µg/mL Col IV at a density of 1 × 105 cells per well, and cultured in 2 mL of ST(2D) medium [DMEM/F12 supplemented with 0.1 mM 2‐mercaptoethanol, 0.5% Penicillin‐Streptomycin, 0.3% BSA, 1% ITS‐X supplement, 2.5 µM Y27632, 2 µM forskolin, and 4% KSR]. The medium was replaced at Day 3, and the cells were analyzed at Day 6.

For the induction of ST(3D)‐TSblast cells, 2.5 × 105 TSblast cells were seeded in 6 cm Petri dishes and cultured in 3 mL of ST(3D) medium [DMEM/F12 supplemented with 0.1 mM 2‐mercaptoethanol, 0.5% Penicillin‐Streptomycin, 0.3% BSA, 1% ITS‐X supplement, 2.5 µM Y27632, 50 ng/mL EGF, 2 µM forskolin, and 4% KSR].

An equal amount of fresh ST(3D) medium was added at Day 3. The cells were passed through a 40 µm mesh filter to remove dead cells and debris at Day 6. We collected and analyzed ST(3D)‐TSCT and ST(3D)‐TSblast cells remaining on the 40 µm mesh filter. Cell lysates were obtained at Day 8 (TS cells) and Day 5 (ST(3D)‐TS cells, ST(2D)‐TS cells) in 1% (v/v) SDS, 50 mM HEPES, pH 8.0, (detail) and quantified by Micro BCA Protein Assay (Thermo Fisher Scientific).

### 4.3 Proteomics: Proteomic Sample Preparation

Samples used for sample preparation and proteome analysis: oocyte (*n* = 5, pooled into five sets from 18 ovums), 8 cell (*n* = 5, pooled into five sets from 22), Blastocyst (*n* = 4, pooled into four sets from 22 blastocysts); cells lysates from: TS (*n* = 4), EVT (*n* = 7), ST(3D)‐TS cell (*n* = 6), TS, ST(2D)‐TS cell (*n* = 7).

For MS‐based proteomics, samples (∼5 µg) were normalized and prepared as described [80] in 50 µL of 50 mM HEPES, pH 8.0, and reduced with 10 mM DTT for 45 min at 50°C followed by alkylation with 10 mM iodoacetamide (IAA, Fluka) for 30 min at 25°C in the dark. The reaction was quenched to a final concentration of 20 mM DTT. Sample digestion was performed according to the single‐pot solid‐phase‐enhanced sample preparation (SP3) method. [51] Briefly, 1 µL of a 50 µg/µL SP3 bead stock (Sera‐Mag SpeedBead carboxylate‐modified magnetic particles; hydrophobic and hydrophobic 1:1 mix, GE Healthcare Life Sciences, Freiburg, Germany) were added to 50 µL of protein extract and 60 µL absolute ethanol (final concentration of 50%) and incubated for 10 min (1000 rpm) at 24°C. Tubes were mounted on a magnetic rack; supernatants were removed, and beads were washed three times with 80% ethanol (200 µL each). Beads were resuspended in 100 µL 50 mM triethylammonium bicarbonate (TEAB, Thermo Fisher Scientific), pH 8.0 and digested overnight with trypsin (1:50 trypsin:protein ratio; Promega, V5111) at 37°C, 1000 rpm. The peptide and bead mixture was centrifuged at 20,000 × *g* for 1 min at 24°C, and the supernatant was collected and acidified to a final concentration of 1.5% v/v formic acid, frozen at −20°C overnight, and dried by vacuum centrifugation. Peptides were resuspended in 0.07% trifluoroacetic acid (Thermo Fisher Scientific), quantified by Fluorometric Peptide Assay (Thermo Fisher Scientific).

### 4.4 Proteomic Liquid Chromatography–Tandem Mass Spectrometry

Peptides were analyzed on a Dionex UltiMate NCS‐3500RS nanoUHPLC coupled to a Q‐Exactive HF‐X hybrid quadrupole‐Orbitrap mass spectrometer equipped with a nanospray ion source in positive mode as described. [80] Peptides were loaded (Acclaim PepMap100 C18 5 µm beads with 100 Å pore‐size, Thermo Fisher Scientific) and separated (1.9‐µm particle size C18, 0.075 × 200 mm, Nikkyo Technos Co. Ltd.) with a gradient of 2%–80% acetonitrile containing 0.1% formic acid over 110 min at 300 nL min^−1^ at 55°C (in‐house enclosed column heater). An MS1 scan was acquired from 350–1650 m/z (60,000 resolution, 3 × 10^6^ automatic gain control (AGC), 128 ms injection time) followed by MS/MS data‐dependent acquisition (top 25) with collision‐induced dissociation and detection in the ion trap (30,000 resolution, 1 × 10^5^ AGC, 60 ms injection time, 28% normalized collision energy, 1.3 m/z quadrupole isolation width). Unassigned precursor ions, charge states, and slightly charged species were rejected, and peptide match was disabled. Selected sequenced ions were dynamically excluded for 30 s. Proteome RAW and parameter data have been deposited to the ProteomeXchange Consortium via the MassIVE integrated data repository and are available via identifier MassIVE MSV000092349.

### 4.5 Proteomic Data Processing and Analysis

Peptide identification and quantification were performed as described previously [80, 81] using MaxQuant (v1.6.14) with its built‐in search engine Andromeda [82]. Tandem mass spectra were searched against *Homo sapiens* (human) reference proteome (74,823 entries) supplemented with common contaminants. Search parameters included carbamidomethylated cysteine as fixed modification and oxidation of methionine and N‐terminal protein acetylation as variable modifications. Data were processed using trypsin/P as the proteolytic enzyme with up to two missed cleavage sites allowed. Peptides were identified with an initial precursor mass deviation of up to 7 ppm and a fragment mass deviation of 20 ppm, at less than 1% FDR on peptide spectrum match (PSM) level employing a target‐decoy approach at peptide and protein levels. Label free quantification (LFQ) algorithm in MaxQuant was used to obtain quantification intensity values and processed using Perseus as described [83]. For Gene Ontology (GO) annotation, protein accession IDs were submitted to DAVID Bioinformatics Resources (https://david.ncifcrf.gov/), [84] and g:Profiler. [85] Hierarchical clustering was performed in Perseus using Euclidean distance and average linkage clustering. R was used for data visualisation (ggplot2, ggpubr packages). Functional enrichment analyses (GO, KEGGs) were performed using DAVID [84] and g:Profiler [85]. Pathway EnrichmentMap analysis was performed using Cytoscape (v3.7.1). [86]

### 4.6 Statistical Analysis

Data were analyzed using GraphPad Prism v8.4.3, with all data pre‐tested for normality. If the data were non‐parametric, a Kruskal‐Wallis with a Tukey's post‐hoc test or Mann–Whitney U analysis was performed. If parametric, one‐way ANOVA with a Tukey's post‐hoc test or an unpaired *t*‐test was performed. All data presented as mean plus/minus standard deviation (mean ± SD). In all analyses, *p < 0.05 is considered statistically significant.

## Conflicts of Interest

The authors declare no conflicts of interest.

## Supporting information




**Supporting file 1**: pmic70017‐sup‐0001‐SuppMat.xlsx

## Data Availability

All MS‐based proteomics data and processed parameter/search outputs have been deposited to the ProteomeXchange Consortium via the MassIVE partner repository and are available via MassIVE with identifier (MSV000092349).
